# Cannabidiol (CBD) for Pain: A Narrative Review of Evidence to Date

**DOI:** 10.7759/cureus.118057

**Published:** 2026-10-17

**Authors:** Robert B Raffa, Joseph V Pergolizzi, Frank Breve, Eugene Vortsman, Giustino Varrassi, Salah N EL-Tallawy

**Affiliations:** 1 School of Pharmacy, Temple University, Philadelphia, USA; 2 Research and Development, Enalare Therapeutics, Mullica Hill, USA; 3 Clinical Research, NEMA Research, Naples, USA; 4 Clinical Pharmacy, Mid Atlantic PharmaTech Consultants, Ventnor City, USA; 5 Emergency Medicine, Northwell Health, New Hyde Park, USA; 6 Research, Fondazione Paolo Procacci, Rome, ITA; 7 Medicine, Minia University, Cairo, EGY; 8 Anesthesia and Pain Management, King Khalid University Hospital, Riyadh, SAU

**Keywords:** analgesic pathways, cannabidiol (cbd), pain, pain management, review

## Abstract

Cannabidiol (CBD), the principal non-intoxicating phytocannabinoid of *Cannabis sativa*, is widely marketed and self-administered for pain relief, yet its regulatory status as an analgesic remains unapproved. This review synthesizes preclinical mechanistic data, human pharmacokinetic data, and clinical trial evidence across major pain conditions in order to summarize the current evidence for, or against, efficacy and safety for pain. A synthesis of published information was conducted using PubMed/MEDLINE, PubMed Central, Cochrane Library, ScienceDirect, and other databases, integrating findings from systematic reviews, meta-analyses, randomized controlled trials (RCTs), and pharmacology reviews on CBD (independent of combination products where possible) and pain of any etiology. Preclinical and mechanistic data support plausible physical analgesic pathways for CBD, including receptor modulation, indirect enhancement of endocannabinoid tone via fatty-acid-binding-protein (FABP) inhibition, and anti-inflammatory effects. However, translation to clinical benefit has been inconsistent. In the largest meta-analysis of cannabinoids for pain, cannabinoids reduced chronic pain and improved sleep quality, but effect sizes fell below predefined minimally important clinical differences, and cannabinoids showed no benefit for acute or cancer pain, while increasing non-serious adverse events. CBD randomized trials in knee osteoarthritis, fibromyalgia, and acute low back pain have consistently failed to separate from placebo, whereas smaller/lower-quality trials and observational surveys report subjective benefit. Safety data increasingly point to dose-dependent hepatotoxicity risk. Despite strong biological plausibility and considerable consumer use, current high-quality evidence does not support CBD monotherapy as an effective analgesic across most pain conditions studied to date. Safety monitoring, particularly of liver enzymes at higher doses, is warranted.

## Introduction and background

Chronic pain affects a substantial proportion of the global population and remains difficult to manage with existing pharmacotherapies, many of which have organ toxicity or abuse risks. This treatment gap, combined with the 2018 United States federal legalization of hemp-derived cannabidiol (CBD) and a parallel global loosening of cannabis scheduling, has driven consumer interest in CBD as a “natural” analgesic; U.S. CBD sales were estimated to have reached billions of dollars annually within a few years of legalization [1]. CBD is one of over 100 phytocannabinoids identified in *Cannabis sativa*. Unlike Δ9-tetrahydrocannabinol (THC), CBD is non-intoxicating and does not produce classical cannabimimetic behavioral effects, and its multimechanistic actions make it attractive for therapeutic development where psychoactivity is not wanted [2-4]. The only CBD product currently approved by a major regulator (United States Food and Drug Administration) is Epidiolex® (Jazz Pharmaceuticals, Dublin), which is indicated not for pain but for seizures associated with Dravet syndrome, Lennox-Gastaut syndrome, and tuberous sclerosis complex.

CBD's analgesic pharmacology is polypharmacological rather than mediated through a single mechanism, which provides multiple potential points of activity but also variable efficacy and adverse effects [1]. A comprehensive pharmacology review concluded that data on CBD's mechanism of action and therapeutic potential are abundant and heterogeneous, with detailed analgesic and anti-inflammatory effects demonstrated across neuropathic pain, inflammatory pain, and osteoarthritis models, with dose- and route-dependent effects on pain, hyperalgesia, and allodynia [1-4]. Preclinical plausibility is therefore well established; the central open question is whether these effects translate into clinically meaningful benefit in humans at doses with a sufficiently large safety margin.

CBD may act as an indirect agonist of cannabinoid receptors through increased endocannabinoid tone, most likely through fatty acid binding protein (FABP) inhibition, while also directly antagonizing the cannabinoid (CB) CB1 receptor [1]; its activity at the CB2 receptor is complex, including both partial agonism and negative allosteric modulation. Because partial-agonist behavior depends on receptor density and the tonic activity of the system, CBD's net effect can vary by tissue and by disease state, a “state-dependent” pharmacology that helps explain heterogeneous clinical results [1]. CBD also interacts with the transient receptor potential (TRP) channel family, particularly TRPV1 [3]. CBD acts as an agonist of the TRPV1 receptor and causes rapid desensitization of that channel, a mechanism believed to underlie nociceptor desensitization and CBD's potential therapeutic effect in neuropathic pain. CBD is an inhibitor of fatty acid binding proteins (FABPs) [2], which transport endogenous cannabinoids (endocannabinoids) such as anandamide (AEA) to their catabolic enzyme (FAAH); inhibiting FABP raises endogenous AEA levels. FABP5 deletion in nociceptors suppresses inflammation- and nerve-growth-factor-mediated TRPV1 sensitization via CB1 receptor signaling, providing a mechanistic bridge between CBD and elevated endocannabinoid tone. TRPV1 activation in peripheral sensory neurons mediates nociception in the ascending pain pathway, whereas TRPV1 activation in the descending pathway (descending noxious inhibitory control, DNIC), involving the rostral ventral medulla and periaqueductal gray, mediates antinociception, illustrating why cannabinoid/vanilloid system effects on pain could be bidirectional depending on the anatomical site of action [4]. Table 1 summarizes the principal targets implicated in CBD's proposed analgesic mechanism, with their functional consequences.

**Table 1:** Proposed molecular targets and mechanisms of action of cannabidiol (CBD) relevant to pain AEA: anandamide; CBD: cannabidiol; CB1: cannabinoid type 1 receptor; CB2: cannabinoid type 2 receptor; CCI: chronic constriction injury; FABP: fatty acid binding protein; PAG: periaqueductal gray; RVM: rostral ventromedial medulla; THC: ∆9-tetrahydrocannabinol; TRPV1: transient receptor potential ankyrin/vanilloid 1.

Target/pathway	Proposed CBD action	Functional consequence	Pain-relevant evidence	Source
CB1 receptor	Negative allosteric modulator / low-affinity antagonist	Dampens excessive CB1 signaling; reduced psychoactivity vs THC	Reduced hyperalgesia in neuropathic pain models	Mlost et al., 2020 [1]
CB2 receptor	Partial agonist; complex modulation including negative allosteric effects	Anti-inflammatory, immune-modulatory action on peripheral nociceptors	Reduced inflammatory pain in preclinical models	Mlost et al., 2020 [1]
FABP (fatty acid binding protein)	Competitive inhibitor	Reduces AEA transport/degradation, raising endocannabinoid tone	Indirect CB1/CB2 agonism; FABP5-dependent analgesia	Bogdan et al., 2022 [2]
TRPV1	Agonist causing rapid desensitization	Reduced nociceptor excitability after initial activation	Reversal of allodynia/hyperalgesia in CCI models	Silva-Cardoso et al., 2021 [3]
Descending pathway (PAG/RVM)	TRPV1-mediated antinociceptive signaling	Enhances descending inhibition of ascending pain signals	Site-dependent effect, opposite of peripheral TRPV1 action	Louis-Gray et al., 2022 [4]

The oral bioavailability of CBD is low (<20%) in animal studies, with extensive first-pass metabolism and renal excretion of metabolites; pharmacokinetic data in humans have been sparse [5]. Bioavailability following smoking is about 30%, with no other route reliably characterized in humans [5]; Cₘₐₓ and AUC increase dose-dependently and are reached more rapidly after smoking/inhalation than after oral or oromucosal administration, with Tₘₐₓ typically 0-4 hours. A 2024 systematic review and meta-regression reported that CBD dose, route of administration, and fed/fasted state are major determinants of pharmacokinetics [6], with oral routes providing higher bioavailability and nanotechnology-based formulations producing a faster onset. The fed state was associated with higher Cₘₐₓ and AUC compared with the fasting state, and a higher proportion of female participants than male participants was associated with lower Tₘₐₓ and higher Cₘₐₓ after oral dosing [6]. Because most over-the-counter CBD products are unformulated oils/isolates with low and variable bioavailability, many negative or null clinical trial results may partly reflect inadequate systemic exposure rather than true pharmacodynamic inactivity, a key caveat when interpreting tests of efficacy. Formulation strategies are being tried to overcome CBD's lipophilicity [7]. Self-emulsifying drug delivery systems (SEDDS), nanoemulsions, and surfactant-based capsules have each demonstrated several-fold increases in Cₘₐₓ and AUC relative to unformulated CBD isolate or the reference oral product [8,9]. Table 2 presents a summary of key pharmacokinetic parameters from clinical trials. 

**Table 2:** Pharmacokinetic parameters of cannabidiol by route of administration AUC: area under the curve; CBD: cannabidiol; C_max_: maximum plasma concentration; PK: pharmacokinetics; SEDDS: self-emulsifying drug delivery system; T_max_: time to maximum plasma concentration.

Route	Oral/systemic bioavailability	T_max_	Key modifying factors	Source
Oral, unformulated (oil/isolate)	~13-19% (animal data); highly variable in humans	Several hours; delayed and variable	Extensive first-pass metabolism; low aqueous solubility	Millar et al., 2018 [5]
Oral, fed vs fasted state	Increased Cmax/AUC with food (high-fat meals)	Longer T_max_ when fasted	Fed status raises both C_max_ and AUC substantially	Moazen-Zadeh et al., 2024 [6]
Smoked/inhaled	~31%	Rapid, within minutes	Bypasses hepatic first-pass metabolism	Millar et al., 2018 [5]
Oromucosal/buccal	Not fully characterized; intermediate exposure	Faster than oral, slower than inhaled	Partial avoidance of first-pass metabolism	Millar et al., 2018 [5]
Topical/transdermal	Primarily local; minimal systemic absorption	Not applicable (local effect)	Distinct PK profile; suited to localized peripheral pain	Xu et al., 2020 [7]
Nanoemulsion/SEDDS formulations	Several-fold increase vs unformulated CBD isolate	Faster onset than standard oral	Enhanced solubility overcomes lipophilicity limitation	Pisak et al., 2025 [8], O'Sullivan et al., 2024 [9]

## Review

Methodology 

This narrative review addresses the mechanistic rationale for CBD as an analgesic, human pharmacokinetics relevant to dosing, clinical trial evidence across major pain phenotypes, and the safety profile to provide healthcare providers and informed consumers who interact with them and seek their advice with a comprehensive, objective, and appropriately analytical evidence map. It draws upon findings from multiple published, peer-reviewed systematic reviews, meta-analyses, and randomized controlled trials. Searches were conducted across PubMed/PubMed Central, the Cochrane Library, ScienceDirect, and other databases using combinations of the terms “cannabidiol”, “CBD”, “pain”, “chronic pain”, “neuropathic pain”, “osteoarthritis”, “fibromyalgia”, “cancer pain”, “pharmacokinetics”, “hepatotoxicity”, and “randomized controlled trial”. Priority was given to (a) existing PRISMA-adherent systematic reviews and meta-analyses, (b) individual randomized controlled trials (RCTs) isolating CBD (rather than THC or THC:CBD combinations) where available, and (c) mechanistic/pharmacology reviews. The literature underlying this narrative review was assembled iteratively. Searches drew on indexed biomedical records, together with publisher-hosted primary sources spanning general medical journals. All searches were conducted against publicly indexed content up to September 2026. Sources were included if they were peer-reviewed original research or peer-reviewed publications in an indexed biomedical or life-science journal, plus foundational sources outside biomedicine. This was not a systematic review; thus, a preregistered protocol, PRISMA flow diagram, or formal risk-of-bias appraisal was not applied, consistent with standard practice for a narrative review. The generative AI app Claude (Sonnet v5, Anthropic, San Francisco) assisted with the identification and presentation of the results of the literature search.

Clinical evidence by pain condition

The design and outcome of key randomized controlled trials of CBD alone (not with THC) for pain is presented in Table 3 and is summarized below.

**Table 3:** Key randomized controlled trials of cannabidiol alone (not with THC) for pain APAP: acetaminophen (paracetamol); CI: confidence interval; DB: double-blind; ED: emergency department; FIQ-R: Revised Fibromyalgia Impact Questionnaire; OA: osteoarthritis; PC: placebo-controlled; PRO: patient-reported outcome; RCT: randomized controlled trial; sEMG: surface electromyography; TID: three times daily; VAS: visual analog scale; VNRS: verbal numerical rating scale; WOMAC: Western Ontario and McMaster Universities Osteoarthritis Index.

Source	Pain	Design	N	CBD dose/route	Comparator	Duration	Primary outcome	Result
Pramhas et al., 2023 [10]	Knee OA	RCT, DB, PC	86	Oral, ~600 mg/day, add-on to APAP	Placebo + APAP	8 weeks	WOMAC pain subscale	No difference (2.5 vs 2.4; P = 0.80)
Vela et al., 2022 [11]	Hand OA/psoriatic arthritis	RCT, DB, PC	129	Synthetic oral, 20–30 mg/day	Placebo	12 weeks	Pain intensity (VAS, mm)	No difference (0.23 mm; P = 0.96)
Rasmussen et al., 2026 [12]	Fibro-myalgia	RCT, DB, PC	200	Oral, 50 mg/day	Placebo	24 weeks	FIQ-R pain intensity	No superiority; placebo numerically favored
Bebee et al., 2021 [13]	Acute low back pain	RCT, DB, PC	100	Single 400 mg oral dose	Placebo + standard care	Single dose	VNRS pain at 2 hours	No difference (6.2 vs 5.8)
Alaia et al., 2022 [14], Alaia et al., 2024 [15]	Post-rotator cuff repair	RCT, DB, PC, multi- center	100	Buccal, 25/50 mg TID	Placebo	14 days (1-year follow-up)	VAS pain	Lower only on day 1; no difference in opioid use or 1-year PROs
Walczynska-Dragon et al., 2024 [16]	TMD/sleep bruxism	RCT, DB, PC, 3-arm	60	Topical/oral gel, 5%/10%	Placebo gel	30 days	VAS pain, sEMG	Significant reduction, both doses

Chronic Pain

Multiple systematic reviews have evaluated CBD alone (not combined with THC) specifically for chronic pain. A PRISMA-adherent review of 12 eligible studies concluded that CBD and THC, both derived from cannabis and having almost identical chemical structures, act on the CB receptor system to produce different effects, with psychoactivity being prominent for THC but minimal or absent for CBD. Theoretically, CBD seems to be a reasonable alternative to opioids for chronic pain since pure CBD is non-intoxicating, although more clinical trials are needed to establish clinical and statistical significance [17]. A subsequent 2023 synthesis-without-meta-analysis review reached a more cautious conclusion: CBD may be useful in treating chronic pain, but findings should be interpreted with caution due to the small number of included studies and heterogeneity arising from different study designs and outcome measures, with more robust studies warranted. The largest and most rigorous meta-analysis of cannabinoids (not CBD alone) for pain, including 65 RCTs and 7,017 participants, found that cannabinoids neither reduced acute or cancer pain nor improved quality of life, but did reduce chronic pain and improve sleep quality; both effect sizes were below the predefined minimal important difference, and cannabinoids increased the risk of non-serious adverse events. The authors concluded that the harmful effects of cannabinoids for pain appear to outweigh the potential benefits [18]. A pharmacology-stratified meta-analysis of 152 RCTs (12,123 participants) across all cannabinoid types found that CBD showed a significant therapeutic effect for epilepsy and parkinsonism, while moderate-grade evidence for chronic pain was found for dronabinol and nabiximols (Sativex®; a buccal spray cannabis extract containing THC and CBD), not for isolated CBD specifically, with all other significant effects rated low, very low, or of no evidential grade [19]. This pharmacology-stratified analysis is important: much of the “cannabis helps chronic pain” literature reflects THC-containing or THC:CBD combination products, not CBD alone.

Osteoarthritis

The most rigorously designed CBD-alone RCT to date targeted knee osteoarthritis (KOA) [10]. In an 86-patient, 8-week, randomized, double-blind, placebo-controlled trial of high-dose oral CBD added to paracetamol, the mean reduction in the WOMAC (Western Ontario and McMaster Universities Osteoarthritis Index) pain subscale was 2.5 (95% CI, 1.8-3.3) in the cannabidiol group versus 2.4 (95% CI, 1.7-3.2) in the placebo group, with no significant between-group difference; adverse events were significantly more frequent with CBD, and increases in liver aminotransferases and gamma-glutamyltransferase above baseline were significantly more common in the CBD group [10]. An earlier, lower-dose (20-30 mg/day), 12-week RCT of synthetic CBD in 136 patients with hand osteoarthritis and psoriatic arthritis (129 analyzed) likewise failed to demonstrate superiority over placebo [11]. Editorial correspondence following the KOA trial emphasized that this was a well-designed RCT addressing many of the shortcomings of prior CBD pain trials and that the only previously published peer-reviewed RCT of oral cannabinoids for OA pain used a much lower CBD dose, underscoring persistent uncertainty about optimal dosing even where trial quality is high.

Neuropathic Pain

A double-blind, crossover RCT of topical CBD oil in symptomatic peripheral neuropathy of the legs is frequently cited as showing a positive signal among patients with symptomatic peripheral neuropathy of at least three months' duration [7], though this trial was small and used a topical route with distinct (largely local, nonsystemic) pharmacokinetics. Broader systematic reviews of cannabinoids for neuropathic pain (mixing CBD-alone, THC-alone, and combination products) generally reported modest, inconsistent benefits, with most positive signals attributable to THC-containing formulations rather than CBD alone [20].

Fibromyalgia

Fibromyalgia has been a particular focus of CBD consumer use, but the highest-quality trial evidence is negative. The CANNFIB trial [12], a 24-week, single-center, randomized, double-blind, placebo-controlled trial of oral CBD 50 mg/day in patients meeting ACR fibromyalgia criteria, found that administration of plant-derived CBD at 50 mg daily for 24 weeks did not demonstrate superiority over placebo in reducing average pain intensity; study findings actually indicated more favorable outcomes for pain and health-related quality of life in the placebo group, although between-group differences were not clinically meaningful [12]. The study authors concluded that the results do not support the use of CBD as an analgesic supplement in fibromyalgia. In contrast, a large (n = 2,701) online survey found that many participants indicated that CBD products were helpful for a broad array of symptoms associated with fibromyalgia and chronic pain, including pain, anxiety, and sleep [21], which illustrates the frequent discordance between self-reported/observational CBD data and randomized trial data, likely influenced by placebo response, expectation effects, and reporting bias.

Acute and Postoperative Pain

The CANBACK (Cannabidiol for Acute Back Pain) trial, a 100-patient RCT of single-dose oral CBD (400 mg) as an adjunct to standard emergency department analgesia for acute nontraumatic low back pain, found comparable pain scores and length of emergency department stay between the CBD and placebo groups at two-hour follow-up [13]. An accompanying editorial concluded that CBD's lack of efficacy for relieving back pain meant it was time to reset expectations [22]. A randomized, placebo-controlled trial of buccally absorbed CBD (25 or 50 mg TID, weight-based) in 100 patients after arthroscopic rotator cuff repair reported a significant reduction in VAS (Visual Analog Scale) pain scores only on postoperative day 1, without deficits in patient-reported outcomes at one-year follow-up [14,15], a narrow, transient effect rather than a robust analgesic signal.

Cancer-Related Pain

Data specific to CBD alone in cancer pain remain limited. The largest cannabinoid meta-analysis found no reduction in cancer pain across included trials, most of which used THC-containing or combination products [18]. Earlier RCTs as an adjunct to opioids in advanced cancer pain showed mixed results and were not attributable to CBD alone.

Other Pain Phenotypes

A three-arm RCT of topical/intraoral CBD gel formulations (5% and 10%) for muscular tension, pain, and sleep bruxism associated with temporomandibular disorders in 60 patients found that a 10% CBD formulation reduced VAS pain by 57.4% and surface electromyographic activity by 42.1%; a 5% formulation reduced pain by 40.8%, with the sleep bruxism index also improving most in the 10% group [16]. This is one of the more positive CBD-alone signals in the literature, though from a single small trial requiring replication. 

Table 4 presents a GRADE-style summary of findings by pain condition.

**Table 4:** Summary of findings by pain condition CANNFIB: cannabidiol for the treatment of fibromyalgia trial; CBD: cannabidiol; DILI: drug-induced liver injury; OA: osteoarthritis; MID: minimal important difference; RCT: randomized controlled trial; THC: ∆9-tetrahydrocannabidiol; TMD: temporomandibular disorder.

Condition	Trials (N)	Direction of effect	Certainty (GRADE)	Key limitation
Chronic pain, general (mixed cannabinoids)	65 RCTs (7,017)	Small reduction, below MID	Low	Combines THC, CBD, nabiximols
Knee osteoarthritis (isolated CBD)	1 RCT (86)	Null	Moderate	Single trial, well-designed
Hand OA/psoriatic arthritis (isolated CBD)	1 RCT (136)	Null	Moderate	Low dose (20–30 mg/day)
Fibromyalgia (isolated CBD)	1 RCT, CANNFIB	Null / placebo favored	Moderate	Single-center, low dose (50 mg)
Acute low back pain (isolated CBD)	1 RCT (100)	Null	Moderate	Single dose only
Postoperative pain (isolated CBD)	1–2 RCTs (~100)	Transient (day 1 only)	Low	Small, limited follow-up
TMD (isolated CBD, topical)	1 RCT (60)	Positive	Low	Single small trial
Cancer pain (mixed cannabinoids)	Subset of 65 RCTs	Null	Low	Mostly THC/nabiximols
Hepatotoxicity/DILI	28 trials (1,533)	Increased risk, dose-dependent	Moderate-high	Causality not fully adjudicated

Safety and adverse effects

Safety has emerged as an increasingly prominent concern as isolated, higher-dose CBD trials have accumulated (Table 5). A systematic review and meta-analysis of 28 clinical trials (n = 1,533) reported that CBD use was associated with an increased probability of liver enzyme elevation (OR 5.85, 95% CI 3.84-8.92) and drug-induced liver injury (OR 4.82, 95% CI 2.46-9.45) compared with placebo; high-dose CBD (≥1,000 mg/day or ≥20 mg/kg/day) and concomitant antiepileptic drug use were identified as risk factors, and no cases were reported in adults using CBD doses below 300 mg/day, nor were any cases of severe drug-induced liver injury reported [23]. The authors concluded that CBD hepatotoxicity was strongly associated with daily high-dose use and concomitant use of antiepileptic drugs, particularly valproate. Follow-up clinical guidance emphasized that CBD-associated liver enzyme elevations and drug-induced liver injury meet the Council for International Organizations of Medical Sciences' classification of a common adverse drug reaction, yet these potential adverse events remain unknown to many clinicians and may be overlooked, prompting a call for structured liver function monitoring protocols [24]. Notably, the 2023 KOA RCT independently replicated this signal at the clinical trial level: liver aminotransferase and gamma-glutamyltransferase levels above baseline were significantly more common in the CBD group than in the placebo group, even outside the epilepsy subpopulation, where hepatotoxicity was first characterized.

**Table 5:** Adverse event and safety signal summary AED: antiepileptic drug; CBD: cannabidiol; CI: confidence interval; DILI: drug-induced liver injury; NS: non-significant; OR: odds ratio; RCT: randomized controlled trial; RR: relative risk.

Adverse event category	Effect estimate (95% CI)	Dose/risk factor	Source
Liver enzyme elevation	OR 5.85 (3.84-8.92)	Any CBD vs placebo, 28 trials	Lo et al., 2023 [23]
Drug-induced liver injury (DILI)	OR 4.82 (2.46-9.45)	Any CBD vs placebo, 28 trials	Lo et al., 2023 [23]
Pooled proportion, liver enzyme elevation	7.4% (4.5-12.1%)	CBD-treated participants, 28 trials	Lo et al., 2023 [23]
Pooled proportion, DILI	3.0% (1.4-6.3%)	CBD-treated participants, 28 trials	Lo et al., 2023 [23]
Non-serious adverse events	RR 1.20 (1.15-1.25)	vs placebo, 65 RCTs	Barakji et al., 2023 [18]
Serious adverse events	RR 1.18 (98% CI 0.95-1.45), NS	vs placebo, 65 RCTs	Barakji et al., 2023 [18]
All-cause mortality	RR 1.20 (98% CI 0.85-1.67), NS	vs placebo, 7 RCTs (2,073)	Barakji et al., 2023 [18]
Lowest-risk dosing threshold	No cases reported	Doses <300 mg/day	Lo et al., 2023 [23]
Key risk factor	Elevated OR	≥1,000 mg/d or ≥20 mg/kg/d; concomitant AED (especially valproate)	Lo et al., 2023 [23]

Across the largest cross-cannabinoid meta-analysis, cannabinoids increased the risk of nonserious adverse events (RR 1.20, 95% CI 1.15-1.25) but not serious adverse events (RR 1.18, 98% CI 0.95-1.45), and none of the included trials reported on cannabinoid dependence or psychosis [18].

Drug interactions. CBD is metabolized via hepatic CYP3A4/CYP2C19 pathways [25], creating a potential risk of interactions with anticoagulants (e.g., warfarin), immunosuppressants (e.g., tacrolimus), opioids (e.g., buprenorphine), and antiepileptics (e.g., valproate, clobazam). 

Regulatory status

Regulatory frameworks for CBD vary substantially by jurisdiction and continue to evolve [26]; readers should verify current rules for their location, as this area changes frequently. As of recent reviews, the Australian Therapeutic Goods Administration [27] has authorized low-dose CBD-containing products, up to a maximum of 150 mg/day, for supply over the counter through pharmacies, while other regulators, including the U.S. FDA, European Food Safety Authority, and UK Committee on Toxicity, have expressed ongoing concerns about safety data gaps, including hepatotoxicity. No regulator has approved CBD specifically as an analgesic; the sole approved indication for prescription CBD (Epidiolex®) remains drug-resistant pediatric epilepsy syndromes [28].

Discussion and limitations

Taken together, the evidence base reveals a striking pattern: strong mechanistic plausibility but weak-to-null clinical trial efficacy for CBD alone across nearly every well-powered, placebo-controlled trial in a distinct pain population, contrasted with more favorable but methodologically weaker observational, survey-based, and small pilot trial data. Several factors likely contribute to this discrepancy.

Pharmacokinetic Underdosing

Given oral bioavailability as low as 13-19% [29], many trials and virtually all consumer over-the-counter products may deliver insufficient systemic CBD exposure to engage the receptor systems characterized in preclinical models. Thus, perceived low efficacy or inactivity in trials might actually reflect insufficient levels needed to produce the biological effect.

THC Conflation

Much of the broader “medical cannabis helps pain” literature involves THC-containing or THC:CBD combination products [30]; when analyses are stratified by specific cannabinoid pharmacology, isolated CBD's evidence for chronic pain drops to low or very-low grade, while dronabinol (THC) and others show comparatively better-supported effects.

Placebo and Expectation Effects

Cannabis-related products carry strong prior expectations of benefit [31], plausibly inflating observational and open-label results relative to blinded RCTs.

Publication and Dosing Heterogeneity

Trial doses range from 20 mg/day to 800+ mg/day, formulations range from unformulated oils to nanoemulsion capsules, and outcome measures vary (WOMAC, VAS, NRS, FIQ-R), limiting direct comparability and meta-analytic power [32].

Regarding safety, the dose-dependent hepatotoxicity association is a clinically important finding that reframes CBD from a presumed “harmless” supplement to a compound requiring the same pharmacovigilance as other hepatically metabolized drugs, particularly at doses above ~300 mg/day or with concomitant antiepileptic use [23].

This review has limitations of its own. It is a high-level narrative synthesis of existing systematic reviews and individual RCTs rather than a de novo, PRISMA-registered systematic review with formal risk-of-bias grading and meta-analytic pooling; it should be read as a relatively comprehensive map of the evidence landscape rather than as a substitute for a fully independent systematic review and meta-analysis. Findings on rapidly evolving topics (e.g., novel high-bioavailability CBD formulations, real-world hepatotoxicity surveillance) may be superseded by newer data; readers should consult the primary sources directly and check for updates in future publications.

## Conclusions

Despite a plausible and richly characterized preclinical mechanism of action spanning TRPV1/TRPA1 modulation, endocannabinoid tone enhancement via FABP inhibition, and anti-inflammatory signaling, the highest-quality randomized controlled trials of isolated cannabidiol conducted to date have largely failed to demonstrate clinically meaningful analgesic superiority over placebo in knee osteoarthritis, fibromyalgia, and acute low back pain, with only narrow or transient positive signals in postoperative and orofacial pain. Broader CBD meta-analyses including THC-containing products show a modest, subclinically important reduction in chronic pain but no benefit in acute or cancer pain, along with an increased risk of nonserious adverse events. A separate and increasingly well-documented safety signal, dose-dependent liver enzyme elevation and drug-induced liver injury, especially at doses above ~300 mg/day or with concomitant antiepileptic drugs, warrants clinical attention regardless of one's view on efficacy. Future research should prioritize adequately powered trials of dose-optimized, bioavailability-enhanced CBD formulations tested against placebo in single, well-defined pain populations, with attention to hepatic safety monitoring.
